# Inhibitory Effects of α-Lipoic Acid on Oxidative Stress-Induced Adipogenesis in Orbital Fibroblasts From Patients With Graves Ophthalmopathy

**DOI:** 10.1097/MD.0000000000002497

**Published:** 2016-01-15

**Authors:** Sena Hwang, Jung Woo Byun, Jin Sook Yoon, Eun Jig Lee

**Affiliations:** From the Department of Internal Medicine (SH, EJL); Brain Korea 21 Plus Project for Medical Science (JWB, EJL); and Department of Ophthalmology, Yonsei University College of Medicine, Seoul, Korea (JSY).

## Abstract

A choice of the optimal treatment for Graves ophthalmopathy (GO) is a challenge due to the complexity of the pathogenesis. Alpha-lipoic acid (ALA) is well known as a multifunctional antioxidant, helping to protect cells against oxidative stress and inflammatory damage.

The aim of this study was to investigate the effects of ALA on intracellular production of reactive oxygen species (ROS), inflammation, and adipogenesis using primary cultured orbital fibroblasts from patients with GO.

Intracellular ROS levels and mRNA expressions of proinflammatory cytokines and chemokines including intercellular adhesion molecule-1 (ICAM-1), interleukin (IL)-6, monocyte chemoattractant protein (MCP)-1, and regulated upon activation normal T cell expressed and presumably secreted (RANTES) were measured. After adipogenesis, the expressions of peroxisome proliferator-activated receptor (PPAR)γ, CCAAT-enhancer-binding proteins (C/EBP)α and β, and heme oxygenase-1 (HO-1) were investigated.

H_2_O_2_ dose-dependently stimulated ROS production and HO-1 expression. Addition of ALA strongly attenuated ROS production and further increased HO-1 expression. However, by pretreatment of zinc protoporphyrin (ZnPP), HO-1 inhibitor, ALA inhibition of ROS generation by H_2_O_2_ was abolished. Tumor necrosis factor (TNF)α-induced mRNA expressions of ICAM-1, IL-6, MCP-1, and RANTES were inhibited by ALA treatment. In this context, TNFα-induced phosphorylation of P65 was also inhibited. In addition, ALA dose-dependently inhibited H_2_O_2_-induced intracellular accumulation of lipid droplets. The expression of adipogenic transcription factors, including PPARγ, C/EBPα, and β, was also inhibited.

ALA is a potential therapeutic agent for GO because of the inhibitory effects on ROS production and gene expression of proinflammatory cytokines and chemokines, resulting in prevention of adipose-tissue expansion.

## Abstract 2

Supplemental Digital Content is available in the text

## INTRODUCTION

Graves ophthalmopathy (GO), the main extrathyroidal manifestation of Graves disease (GD), has large indirect and direct effects on health system.^1,2^ GO includes ocular involvement and is clinically relevant to 25% to 50% of patients with Graves GD, and approximately 5% of them have vision-threatening compressive optic neuropathy.^1^ Although the precise pathogenesis of GO remains unclear, the volume expansion of the orbital tissue due to excess accumulation of glycosaminoglycans and adipogenesis of orbital fibroblasts are the key pathological features of GO.^2,3^ These processes are thought to be driven in GO, at least in part, by oxidative stress.

A number of studies provided evidence that increased oxidative stress plays an important pathogenic role in GO.^4^ Oxidative free radicals stimulate proliferation of GO-affected orbital fibroblasts and lead to excessive production of glycosaminoglycans.^5,6^ In addition, oxidative stress significantly enhances differentiation to adipocytes of primary cultured orbital preadipocytes from patients with GO.^7^ These GO-related orbital fibroblasts include a higher proportion of preadipocytes capable of adipocyte differentiation compared with normal fibroblasts, thus, this unique signature could also contribute to the expansion of orbital tissue volume by adipogenesis.^8,9^ In an earlier study, we found that cigarette smoke extract and hydrogen peroxide (H_2_O_2_) significantly upregulated intracellular reactive oxygen species (ROS) concentration in adipocyte differentiating orbital fibroblasts from patients with GO, and these changes are attenuated by quercetin, which has antioxidant properties.^10^ In addition, oxidative stress can trigger an inflammatory cytokine and chemokine cascade through nuclear factor-kappaB (NF-κB) activation.^11^ Overall, several evidences so far suggest that oxidative stress, inflammation, and connective tissue remodeling—the major pathogenic processes of GO—are closely linked.

Although a choice of the optimal treatment is a challenge due to the complexity of the pathogenesis, antioxidant agents may serve as a preventive strategy against the development and progression of GO according to several in vitro and clinical studies.^12–14^ In this context, we further studied the role of oxidative stress in the pathophysiology of GO in the present study, and suggest that a powerful antioxidant agent, alpha-lipoic acid (1,2-dithiolane-3-pentanoic acid; ALA), may have therapeutic benefits targeting oxidative stress, inflammation, and adipogenesis in GO.

## METHODS

### Tissue Collection, Cell Culture, and the Adipogenesis Protocol

Orbital adipose/connective tissues were collected from surgical specimens of 7 patients with GO during decompression (surgical treatment) of severe proptosis (4 men and 3 women; mean age 39.8 years) (Table 1).^15,16^ All patients were euthyroid at the time of surgery and had not been treated with glucocorticoid or radiation for at least 3 months. Normal control fat tissues were obtained from 6 individuals without a history of GO or autoimmune thyroid disease who underwent surgical treatment for noninflammatory conditions (2 men and 4 women; mean age 41.0 years). All of the patients provided informed consent, and the study protocol was approved by the institutional review board of Severance Hospital.

**TABLE 1:**
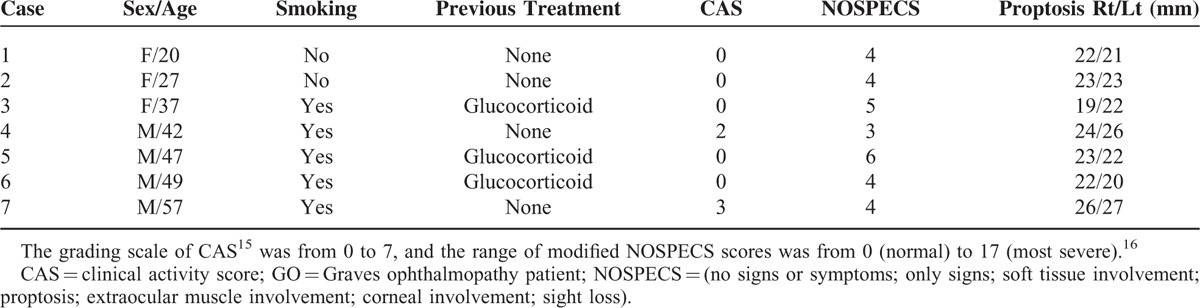
Clinical Characteristics of Patients in the Study

The protocol of cell culture and adipocyte differentiation was described previously.^10,14^ Orbital tissue explants were minced and placed in plastic culture dishes containing the explant medium (high-glucose DMEM, 20% fetal bovine serum [FBS], 100 U/mL penicillin, and 20 μg/mL gentamycin) at 37°C in a humidified incubator containing 5% of CO_2_. After the orbital fibroblasts grew out of the explants, these fibroblasts were cultivated and passaged in 10-cm culture dishes containing the growth medium (DMEM plus 10% FBS). The cells were used for experiments between the third and fifth passages.

Orbital preadipocyte fibroblasts were induced to differentiate into adipocytes for 10 days by the following the protocol. In brief, the cells were grown in 6-well plates or 6-cm culture dishes, then exposed to the adipogenic cocktail (DMEM, 10% FBS, 33 μM biotin, 17 μM pantothenic acid, 10 μg/mL transferrin, 0.2 nM T3, 0.2 μM cPGI2, and 1 μM insulin). For the first 4 days, 1 μM dexamethasone and 0.1 mM isobutylmethylxanthine were included in the adipogenic cocktail, and the media were replaced at day 0 and day 2. After day 4, the media was replaced every 3 days. For further stimulation of the differentiation, 10 μM rosiglitazone alone or 10 μM rosiglitazone plus 10 μM H_2_O_2_ were added in the adipogenic cocktail when the media was replaced during adipogenesis from day 1 to day 10. Rosiglitazone was purchased from Cayman Chemical (Ann Arbor, MI).

### Oil Red O Staining for Analysis of Adipogenesis

After the adipogenic differentiation, cytoplasmic lipid droplets were stained with Oil Red O to visualize the lipids as described previously.^10,14^ The intracellular lipid droplets were examined under an Axiovert 35 light microscope (Carl Zeiss, Thornwood, NY) and photographed at ×40 and ×400 magnifications with an Olympus BX60 light microscope (Olympus, Melville, NY). For quantitation of the lipid droplets, the cell samples were dried at room temperature, and cell-bound Oil Red O was solubilized with 100% isopropanol. The solubilized dye was quantitated on a spectrophotometer at 490 nm.

### Cell Viability Assays

The effect of ALA on viability of orbital fibroblasts was analyzed by the (3-(4,5-dimethylthiazol-2-yl)-5-(3-carboxymethoxyphenyl)-2-(4-sulfophenyl)-2H-tetrazolium, inner salt) (MTS) assay. Orbital fibroblasts from normal controls and patients with GO were seeded in 96-well culture plates (10^4^/well) and incubated with various concentrations of 0 to 1000 μM ALA (Sigma-Aldrich, St. Louis, MO) for 24, 48, or 72 h in the CO_2_ incubator at 37°C. After that, 20 μL of the MTS reagent was added to every well and incubated for 4 h. Absorbance of the plates was measured at 490 nm on a microplate reader (Bio-Tek Instruments, Winooski, VT). Cell viability was expressed as the percentage of untreated control cells. Based on the MTS assay, the 0 to 1000 μM range of ALA did not affect cell viability and did not cause morphological changes (Supplemental Data).

### Quantification of Intracellular ROS

Intracellular ROS levels were determined by means of the fluorescent dye 5-(and 6)-carboxy-2′,7′-dichlorodihydrofluorescein diacetate (H_2_DCFDA; Invitrogen, Eugene, OR), which is converted into a membrane-penetrating and highly fluorescent reagent, DCF (2′,7′-dichlorofluorescin), in the cell in the presence of ROS. To assess the effects of ALA on ROS production, the cells were pretreated with 0, 100, 250, or 500 μM ALA for 24 h. After that, the cells were incubated with 10 μM H_2_DCFDA in PBS for 30 min at 37°C, and then stimulated with 100 μM H_2_O_2_ in PBS for 30 min at 37°C. Finally, the cells were collected, and fluorescence intensity was measured by a flow cytometry (FACSverse, BD Biosciences, Franklin Lakes, NJ).

### RNA Isolation and Real-Time RT-PCR

The cells were grown to confluence in 6-cm dishes, then culture media was changed to serum-free DMEM, and incubated (24 h) with 10 ng/mL tumor necrosis factor (TNF)α (R and D Systems, Minneapolis, MN) and 10 ng/mL interferon (IFN)γ (R and D Systems, Minneapolis, MN), alone or in combination. The concentration of TNFα and IFNγ was selected on the basis of the previous report.^17^ To investigate the effect of ALA on TNFα-induced cytokines and chemokines, cells were stimulated with TNFα (10 ng/mL) for 24 h in the absence or presence of concentrations of ALA (100, 250, or 500 μM).

Purified RNA was prepared and then reverse-transcribed into single-stranded cDNA according to the manufacturer's instructions. The cDNA was amplified by quantitative real-time PCR (RT-PCR) on an ABI 7300 real-time PCR thermocycler (Applied Biosystems, Carlsbad, CA) with CYBER Green. The primers were designed from available human gene sequences as follows: intercellular adhesion molecule (ICAM)-1, forward 5′-GGCCTCAGCACGTACCTCTA-3′ and reverse 5′-TGCTCCTTCCTCTTGGCTTA-3′; interleukin (IL)-6, forward 5′-TTGGCAGCCTTCCTGATTTC-3′ and reverse 5′-AACTTCTCCACAACCCTCTG-3′; monocyte chemoattractant protein (MCP)-1, forward 5′- ATGCAATCAATGCCCCAGTC-3′ and reverse 5′- TGCAGATTCTTGGGTTGTGG-3’; regulated upon activation normal T cell expressed and presumably secreted (RANTES), forward 5′- CAGTCGTCTTTGTCACCCGAA-3′ and reverse 5′- TCCCAAGCTAGGACAAGAGCA-3′; and GAPDH, forward 5′-AGGGCTGCTTTTAACTCTGGT-3′ and reverse 5′-CCCCACTTGATTTTGGAGGGGA-3′. The comparative Ct method (threshold cycle where amplification is in the linear range of the amplification curve) was used for relative quantification of the gene expression. These data were normalized to GAPDH and then calculated as 2^–ΔΔCt^ relative to the control.

### Western Blotting

The cells were lysed for western blot analysis using lysis buffer, and the western blot was performed as described previously.^10,14^ Immunoblot analyses were performed with mouse anti-peroxisome proliferator-activated receptor (PPAR) γ (#sc-7273, 1:1000), rabbit anti-CCAAT-enhancer-binding proteins (C/EBP)α (#sc-61, 1:500), rabbit anti-C/EBPβ (#sc-150, 1: 2000), rabbit anti-heme oxygenase (HO)-1 (#sc-10789, 1:1000), and mouse β-actin (#sc-47778, 1:10000) that were all obtained from Santa Cruz Biotechnology (Santa Cruz, CA). Rabbit phospho-NF-κB p65 (Ser536) (#3034, 1:1000) and rabbit NF-κB p65 (D14E12) (#8242, 1:1000) were purchased from Cell Signaling Technology, Inc. (Boston, MA). Immunoreactive bands were detected by means of the Enhanced Chemiluminescence Detection Kit (Amersham Pharmacia Biotech), and X-ray film was exposed to the bands (Amersham Pharmacia Biotech). The signals were quantified by densitometry and normalized to the β-actin level in the same sample.

### Statistical Analysis

All experiments were performed at least 3 times on samples from different patients, and the results were expressed as mean ± SEM. Statistical significance was analyzed by Student *t* test in the PASW Statistics software, version 18 for Windows. A *P* value <0.05 was assumed to denote statistical significance.

## RESULTS

### Effect of ALA on ROS Production in Orbital Fibroblasts

**FIGURE 1:**
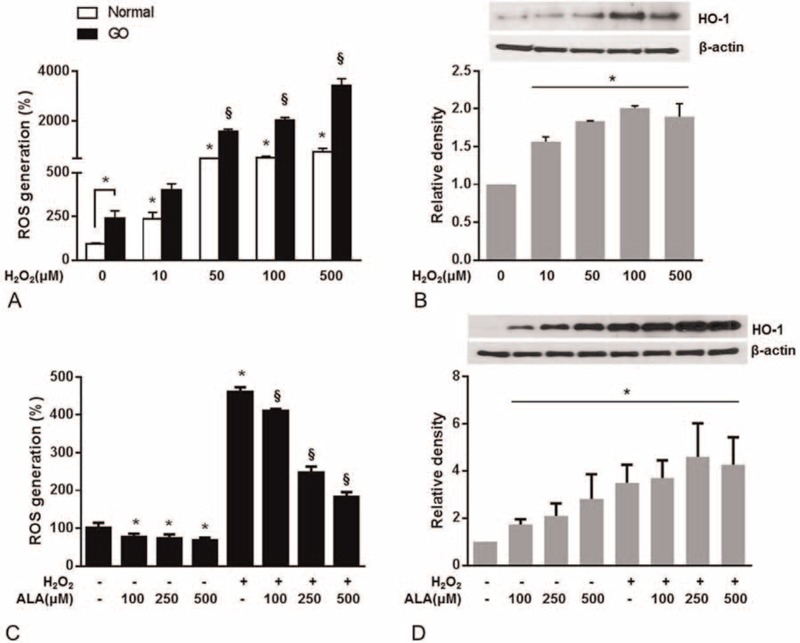
Effects of α-lipoic acid (ALA) on H_2_O_2_-induced intracellular production of reactive oxygen species (ROS) and HO-1 expression in fibroblasts from patients with Graves ophthalmopathy (GO). A, Intracellular ROS were quantified by flow cytometry with 5-(and 6)-carboxy-2′,7′-dichlorodihydrofluorescein diacetate (H_2_DCFDA) in healthy and GO-related cells stimulated with 10 to 500 μM H_2_O_2_ for 30 min. B, HO-1 protein expression was determined by western blotting in GO-related orbital fibroblasts stimulated with 0 to 500 μM H_2_O_2_ for 24 h. C, The GO-related orbital fibroblasts were treated with ALA (100, 250, or 500 μM) for 24 h and then stimulated with 100 μM H_2_O_2_ for 30 min. ROS were quantified by flow cytometry with H_2_DCFDA. D, HO-1 expression in the GO-related cells was analyzed by western blotting. The GO-related cells were treated with ALA (100, 250, or 500 μM) for 24 h with or without 100 μM H_2_O_2_. The results are expressed as a percentage of no-treatment control values, mean ± SEM (n = 3). ^∗^*P* < 0.05 compared with untreated cells; ^§^*P* < 0.05 compared with cells stimulated with H_2_O_2_.

The basal level of intracellular ROS was significantly higher in orbital fibroblasts from patients with GO than in normal control orbital fibroblasts (*P* = 0.024; Figure 1A). Intracellular ROS levels were significantly increased by H_2_O_2_, but this effect was much greater in the GO-related orbital fibroblasts than in normal orbital fibroblasts. Consistent with this result, expression of the HO-1 protein was significantly increased by H_2_O_2_ stimulation for 24 h in a dose-dependent manner (Figure 1B). However, pretreatment with ALA for 24 h, before incubation with or without H_2_O_2_, dose-dependently attenuated this increase in intracellular ROS levels (Figure 1C). Furthermore, the H_2_O_2_-enhanced expression of HO-1 was potentiated by 100, 250, and 500 μM ALA (Figure 1D).

**FIGURE 2:**
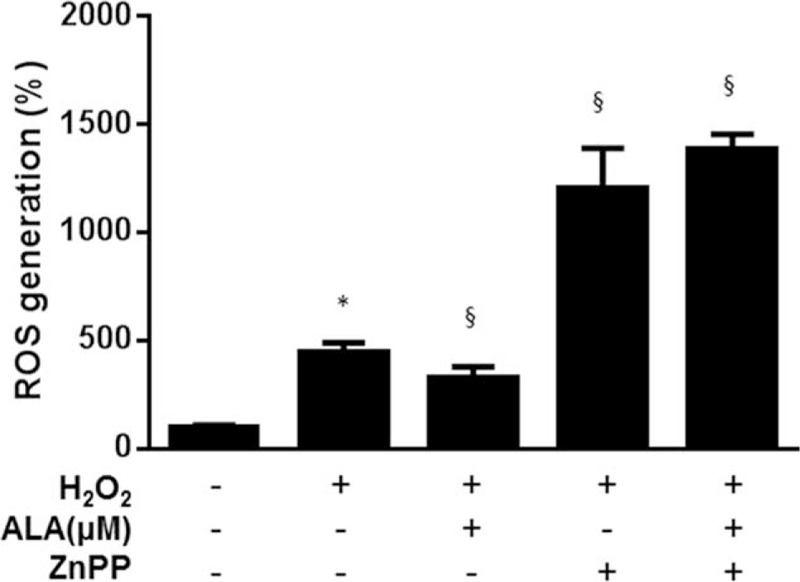
A heme oxygenase 1 (HO-1) inhibitor, zinc protoporphyrin (ZnPP), abrogated the inhibitory effect of α-lipoic acid (ALA) on production of reactive oxygen species (ROS). Orbital fibroblasts from patients with Graves ophthalmopathy (GO) were pretreated with ZnPP (10 μM) for 1 h followed by treatment with ALA (500 μM) for 24 h, and were then stimulated with 100 μM H_2_O_2_ for 30 min. ROS were quantified by flow cytometry with 5-(and 6)-carboxy-2′,7′-dichlorodihydrofluorescein diacetate (H_2_DCFDA). The data are expressed as percentages of the no-treatment control values, mean ± SEM (n = 3). ^∗^*P* < 0.05 compared with the untreated cells; ^§^*P* < 0.05 compared with the cells stimulated with H_2_O_2_.

To determine the possible role of HO-1 in the reduction of the ROS level by ALA in the GO-related orbital fibroblasts, we blocked the HO-1 activity using a selective HO-1 inhibitor, zinc protoporphyrin (ZnPP; Sigma-Aldrich). When the GO-related cells were pretreated with 10 μM ZnPP for 1 h, ALA did not prevent the effect of H_2_O_2_ exposure, indicating that antioxidant effect of ALA was probably through HO-1. During incubation with the HO-1 inhibitor, H_2_O_2_ potentiated the increase in intracellular ROS levels (Figure 2).

### Effects of ALA on TNF-α-Induced Proinflammatory Cytokines and Chemokines

**FIGURE 3:**
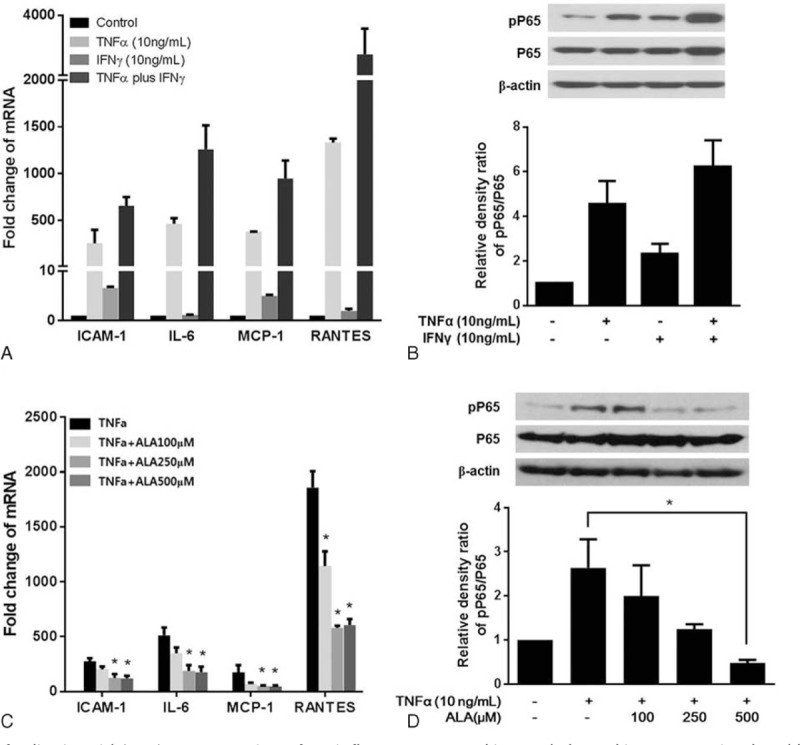
Effects of α-lipoic acid (ALA) on expression of proinflammatory cytokine and chemokine genes stimulated by TNF-α. A, The cells were incubated with 10 ng/mL TNF-α and 10 ng/mL IFNγ, alone or in combination for 24 h. The mRNA levels were determined by RT-PCR, and the results were normalized to the housekeeping gene, GAPDH, and expressed in arbitrary units relative to the levels of no-treatment control, set to 1.0. B, Phosphorylation of P65 (pP65) in the whole-cell extract was determined by western blotting. Quantification of pP65 and P65 by densitometry, normalized to the level of β-actin in the same sample, is shown. C, The cells were incubated with ALA (0, 100, 250, or 500 μM) and 10 ng/mL TNF-α for 24 h. The mRNA levels were determined by RT-PCR. D, The protein expression of pP65 was determined by western blotting. The data in the columns are mean relative density ratios ± SEM (n = 3). ^∗^*P* < 0.05 compared with the cells stimulated with 10 ng/mL TNF-α.

We used RT-PCR to examine gene expression of ICAM-1, IL-6, MCP-1, and RANTES in the absence or presence of ALA (100, 250, or 500 μM) in the GO-related orbital fibroblasts (Figure 3). TNFα (10 ng/mL) simulated mRNA expression of ICAM-1, IL6, MCP-1, and RANTES, but IFNγ (10 ng/mL) alone had no effect on stimulation of IL-6 and RANTES (Figure 3A). The combination of TNFα and IFNγ had a significant synergistic effect on mRNA expression of cytokines and chemokines including ICAM-1, IL6, MCP-1, and RANTES (Figure 3A) and also strongly enhanced the expression of phopho-P65 (pP65) (Figure 3B). Based on these results, TNFα (10 ng/mL) alone seems to be enough to stimulate mRNA expression of these cytokines and chemokines in GO orbital fibroblasts. However, stimulated mRNA expression by TNFα was attenuated with ALA treatment in a dose-dependent manner (significantly at 250 and 500 μM ALA; Figure 3C). In this context, TNF-α (10 ng/mL) induced pP65 in the GO-related orbital fibroblasts, but ALA dose-dependently attenuated this effect with significance at 500 μM (Figure 3D). After pretreatment a nuclear factor kappa B (NF-κB) inhibitor, MG-132 (10 μM for 1 h) before ALA treatment, simulated mRNA expression levels of these cytokines and chemokines were significantly suppressed (96, 98, 98, and 99% decreases for ICAM-1, IL-6, MCP-1, and RANTES, respectively), suggesting this anti-inflammatory effect of ALA was regulated, at least in part, by NF-κB pathway.

### Effect of ALA on Oxidative-Stress-Induced Adipogenesis in the GO-Related Fibroblasts

**FIGURE 4:**
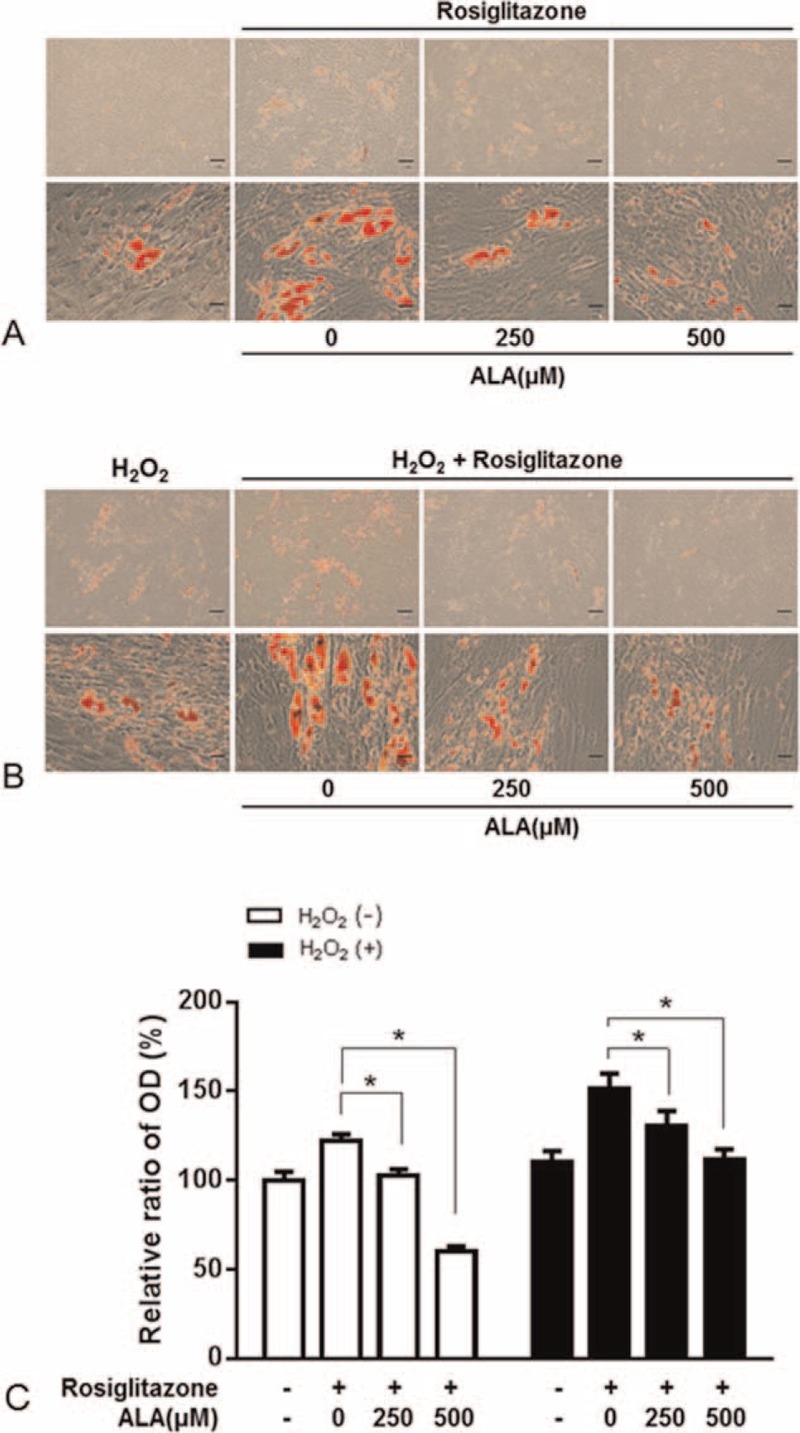
The effect of α-lipoic acid (ALA) on adipogenesis of orbital fibroblasts from patients with Graves ophthalmopathy (GO). Treatment with ALA (250 or 500 μM) for the first 3 days after initiation of the 10-day adipogenesis procedure in the adipogenic medium with or without 10 μM rosiglitazone (A) or its combination with H_2_O_2_ (B). The cells were stained with Oil Red O and examined macroscopically and microscopically (×40 and ×400 magnifications). C, Cell-bound Oil Red O was solubilized, and OD at 490 nm was measured to quantify the adipogenesis. The data in the column are the mean relative density ratios ± SEM (n = 3). ^∗^*P* < 0.05 compared with the cells that differentiated under the influence of rosiglitazone or its combination with H_2_O_2_ (10 μM).

The effect of ALA on adipogenic differentiation of GO-related orbital fibroblasts was examined by Oil Red O staining (Figure 4). The lipid droplets were visible since day 3, and then increased in number and size during adipogenesis. The addition of 10 μM rosiglitazone increased adipogenesis (Figure 4A), and additional H_2_O_2_ treatment along with rosiglitazone during adipogenesis stimulated further accumulation of lipid droplets (Figure 4B). Oil Red O staining showed that ALA dose-dependently decreased the number and size of intracellular lipid droplets compared with the control as demonstrated by morphological and quantitative analysis (Figure 4C).

**FIGURE 5:**
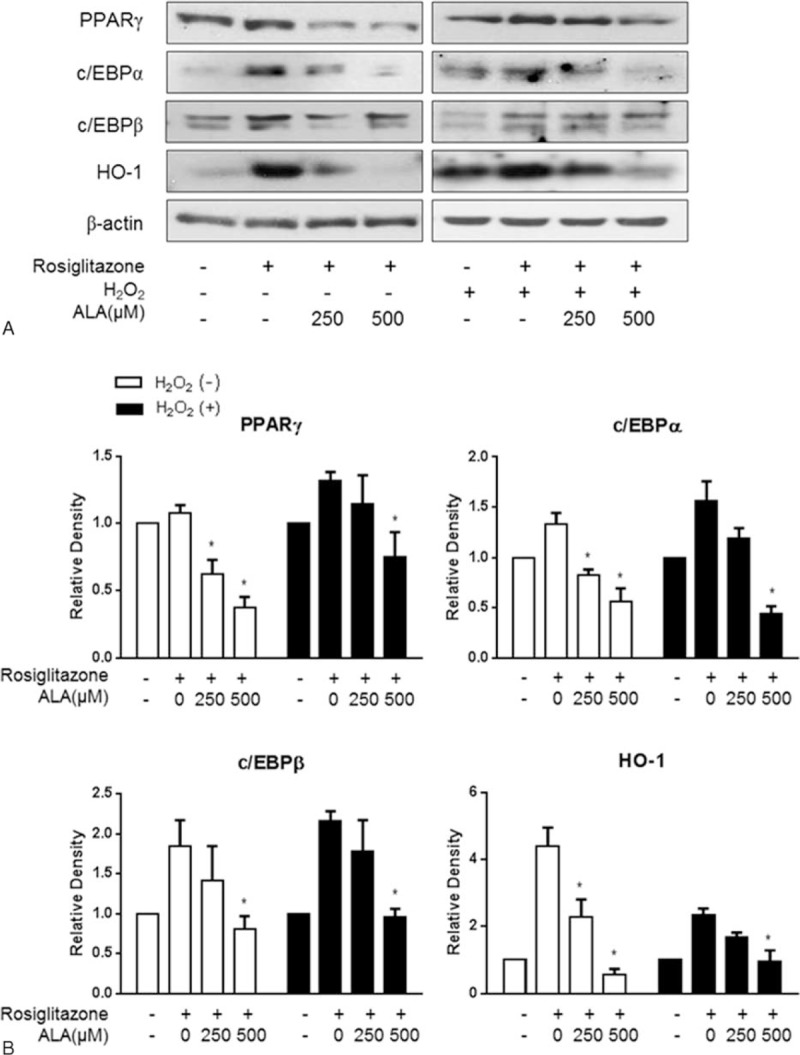
Effects of α-lipoic acid (ALA) on the expression of adipogenic transcriptional regulators during adipogenesis of orbital fibroblasts from patients with Graves ophthalmopathy (GO). Western blot analysis of protein expression of PPARγ, c/EBPα, c/EBPβ, and HO-1 (A). Quantification by densitometry, after normalization to the β-actin level in the same sample, is shown for PPARγ (B), c/EBPα (C), c/EBPβ (D), and HO-1 (E). The data in the column are mean relative density ratios ± SEM (n = 3). ^∗^*P* < 0.05 compared with the cells that differentiated under the influence of rosiglitazone or its combination with H_2_O_2_ (10 μM).

Correlated with these results, ALA dose-dependently and significantly reduced the expression of adipogenic transcription factors, PPARγ, C/EBPα, and C/EBPβ which were stimulated by 10 μM rosiglitazone or its combination with 10 μM H_2_O_2_ (Figure 5A). Notably, HO-1 expression was strongly upregulated by rosiglitazone or its combination with H_2_O_2_ but was attenuated by ALA (Figure 5B).

## DISCUSSION

Although the exact etiology of GO remains unclear, the pathogenesis is being increasingly elucidated. A volume expansion of fatty connective tissues within the bony orbit is linked to most orbital complications. The GO is characterized by marked infiltration of activated T cell producing proinflammatory cytokines that induce glycosaminoglycan production and adipogenic differentiation of orbital fibroblasts, leading to orbital fibrosis and edema.^14,18,19^ Similarly, cigarette smoke extract (CSE) has been shown to significantly stimulate hyaluronan production and adipogenesis in GO orbital fibroblasts.^7,10^ Cigarette smoking is the most important known risk factor of GO and may act in part by upregulation of oxidative stress.^20^

In the present study, oxidative stress stimulated ROS production dose dependently in GO-related orbital fibroblasts, and HO-1 expression was enhanced as a part of the protective response to oxidative stress. ALA, however, significantly inhibited the ROS production via HO-1 and potentiated HO-1 expression after H_2_O_2_ stimulation. Previously, we reported a cigarette smoke extract similarly stimulated ROS production and HO-1 expression.^10^ Recently, a multicenter randomized controlled trial that was conducted by the European Group on GO (EUGOGO) showed that selenium improves sign and symptoms of mild GO.^21^ The mechanism of this effect may involve the remarkable anti-oxidative, anti-inflammatory, and immune modulatory effects of selenium.^22^

ALA is a multifunctional antioxidant. In fact, ALA and its reduced form dihydrolipoic acid (DHLA) have direct scavenging effects on ROS and chelate redox-active transition metals, and regenerate endogenous antioxidants, including vitamin C, vitamin E, and glutathione.^23^ Moreover, ALA has many biochemical functions related to modulation of signal transduction pathways, like insulin, NF-κB, and adenosine monophosphatase protein kinase (AMPK) pathways.^24^ Because of these pleotropic effects of ALA, it has been successfully used against various diseases such as diabetes and its complications, hypertension, Alzheimer disease, and cancers.^25–29^

The induced oxidative stress activates various inflammatory pathways. ROS may stimulate the NF-κB pathway, which plays a key role in the immune response and inflammation which also associated with upregulation of proinflammatory cytokines.^30^ These cytokines are produced predominantly by T cells infiltrating orbital connective tissues and are likely to drive cell activation, leading to orbital connective tissue remodeling.^31^ Many studies have reported that several cytokines and chemokines such as ICAM-1, IL-6, and MCP-1 are stimulated by CD40 ligand in orbital fibroblasts from patients with GO.^32,33^ Recently, it has been reported that RANTES, a T lymphocyte chemoattractant, is highly activated by IL1β in orbital fibroblasts.^34^ Similarly, we found that basal mRNA expression of RANTES was weak, but strongly enhanced by TNFα or TNFα plus IFNγ in our study. The combination of these cytokines, TNFα plus IFNγ, simulates Graves thyrocytes and orbital fibroblasts to produce CXC alpha-chemokine CXCL10/IP-10 which plays an important role in the initiation of GO.^35^ These observations suggest that retrobulbar cell types from GO patients participate in self-perpetuation of inflammation by releasing chemokines under the influence of cytokines.

Several studies have already shown anti-inflammatory properties of ALA in several experimental or clinical settings. ALA inhibits the NF-κB pathway probably due to the inhibitory effect on degradation of IκB through the mitogen-activated protein kinases (MAPK) pathway.^36^ Furthermore, ALA can help regenerate vitamin E, thereby inhibiting protein kinase C that can phosphorylate IκB.^24^ In this study, we found that mRNA expression of cytokines and chemokines including ICAM-1, IL-6, MCP-1, and RANTES induced by TNFα was attenuated by ALA in GO orbital fibroblasts. The mechanism likely involves the inhibition of the NF-κB pathway.

ALA is also a promising antiobesity drug due to its inhibitory effects on adipogenesis. ALA attenuates adipogenic differentiation of 3T3-L1 preadipocytes by modulating expression of adipogenic transcription factors through several signaling pathways such as the MAPK and AMPK pathways.^37,38^ In the present study, we also showed antiadipogenic effects of ALA in GO-related orbital fibroblasts under oxidative stress because we observed down regulation of adipogenic transcription factors, PPARγ, c/EBPα, and c/EBPβ, under the influence of ALA. Meanwhile, HO-1 expression was decreased by ALA after adipogenesis, while this expression was stimulated by H_2_O_2_ and potentiated by ALA in GO-related preadipocytes. These findings suggest underlying the anti-adipogenic effects of ALA might be independent of its antioxidant action.

Taken together, we identified that ALA could be a therapeutic agent because of its inhibitory effects on ROS production, inflammation, and adipose tissue expansion in GO-related orbital fibroblasts. Given the pleiotropic actions of ALA, its therapeutic potential in GO seems promising.

## Supplementary Material

Supplemental Digital Content
