# Supplementary material for: Inhibitory Effects of α-Lipoic Acid on Oxidative Stress-Induced Adipogenesis in Orbital Fibroblasts From Patients With Graves Ophthalmopathy
Source: Medicine (Baltimore). 2016 Jan 15;95(2):e2497. doi: 10.1097/MD.0000000000002497 (PMC4718288; doi:10.1097/MD.0000000000002497)
Supplement: Supplemental Digital Content [file medi-95-e2497-s001.pdf]

Inhibitory effects of α-lipoic acid on oxidative stress-induced adipogenesis in orbital fibroblasts from patients with Graves' ophthalmopathy

Sena Hwang, MD, PhD, Jung Woo Byun, BA, Jin Sook Yoon, MD, PhD, and Eun Jig Lee, MD, PhD.

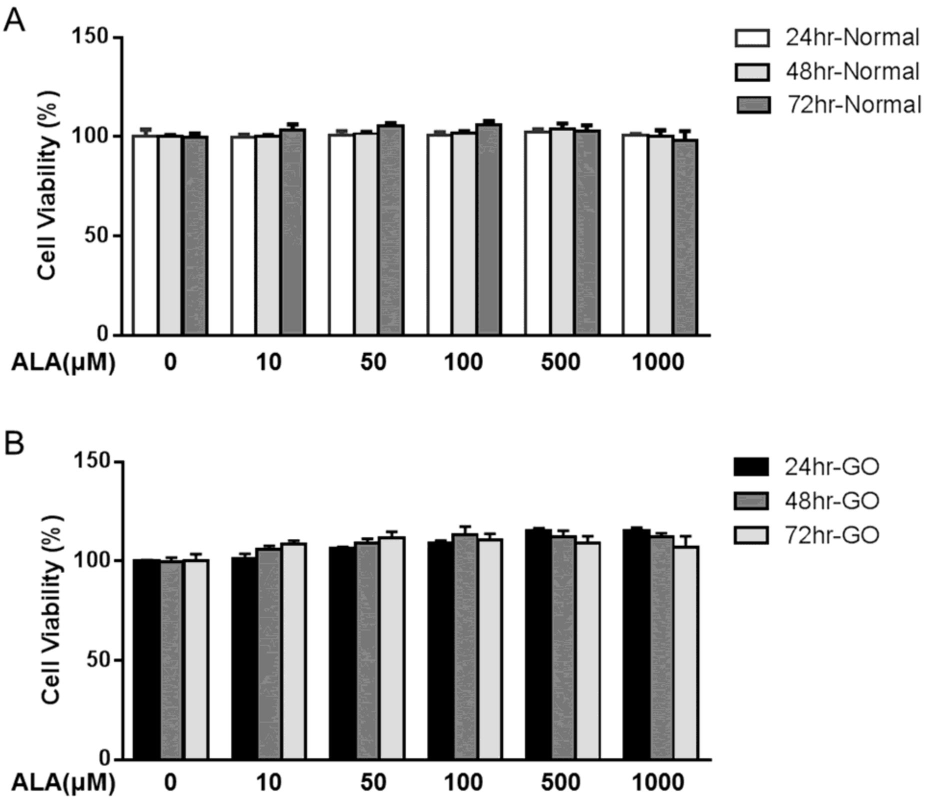

Supplemental Digital Content.

Supplemental figure 1. The effect of α-lipoic acid (ALA) on cell viability in orbital fibroblasts according to the MTS assay.
